# WDR43 is a potential diagnostic biomarker and therapeutic target for osteoarthritis complicated with Parkinson’s disease

**DOI:** 10.3389/fncel.2022.1013745

**Published:** 2022-11-07

**Authors:** Hongquan Heng, Jie Liu, Mingwei Hu, Dazhuang Li, Wenxing Su, Jian Li

**Affiliations:** ^1^Department of Orthopedics, The Second Affiliated Hospital of Soochow University, Suzhou, China; ^2^Department of Plastic and Burn Surgery, The Second Affiliated Hospital of Chengdu Medical College, China National Nuclear Corporation 416 Hospital, Chengdu, China; ^3^Department of Orthopedics, Liyang People’s Hospital, Liyang, China; ^4^Department of Orthopedics, The Affiliated Hospital of Yangzhou University, Yangzhou University, Yangzhou, China; ^5^Department of Neurology, The First Affiliated Hospital of Anhui Medical University, Hefei, China

**Keywords:** osteoarthritis, Parkinson’s disease, weighted gene co-expression network analysis, immune cell infiltration, hub gene

## Abstract

Osteoarthritis (OA) and Parkinson’s disease (PD) are on the rise and greatly impact the quality of individuals’ lives. Although accumulating evidence indicates a relationship between OA and PD, the particular interactions connecting the two diseases have not been thoroughly examined. Therefore, this study explored the association through genetic characterization and functional enrichment. Four datasets (GSE55235, GSE12021, GSE7621, and GSE42966) were chosen for assessment and validation from the Gene Expression Omnibus (GEO) database. Weighted Gene Co-Expression Network Analysis (WGCNA) was implemented to determine the most relevant genes for clinical features. Then, Gene Ontology (GO) and Kyoto Encyclopedia of Genes and Genomes (KEGG) were carried out to explore the biological processes of common genes, and to display the interrelationships between common genes, the STRING database and the application Molecular Complex Detection Algorithm (MCODE) of Cytoscape software were leveraged to get hub genes. By intersecting the common genes with the differentially expressed genes (DEGs) acquired from GSE12021 and GSE42966, the hub genes were identified. Finally, we validated the diagnostic efficacy of hub genes and explored their correlation with 22 immune infiltrating cells. As a consequence, we discovered 71 common genes, most of which were functionally enriched in antigen processing and presentation, mitochondrial translation, the mRNA surveillance pathway, and nucleocytoplasmic transport. Furthermore, WDR43 was found by intersecting eight hub genes with 28 DEGs from the two validation datasets. Receiver Operating Characteristic (ROC) implied the diagnostic role of WDR43 in OA and PD. Immune infiltration research revealed that T-cell regulatory (Tregs), monocytes, and mast cells resting were associated with the pathogenesis of OA and PD. WDR43 may provide key insights into the relationship between OA and PD.

## Introduction

OA is a progressive and degenerative joint disease, which is a chronic disease and disability caused by various pathological changes such as synovial inflammation, cartilage degradation, and subchondral bone changes (McDonough and Jette, 2010; Neogi, 2013). OA has been reported in 10 percent of men and 18 percent of women over the age of 60 (Roseti et al., 2019). This degenerative disease primarily affects the weight-bearing joints of the lower extremities (hips, knees, and ankles). Patients with OA have a reduced quality of life due to limited joint mobility, pain, swelling, and deformity (Ma et al., 2014). A complex interplay between many genetic and environmental risk factors (developmental disorders, obesity, metabolic factors, and pre-existing joint damage) accelerates the onset and progression of OA (Egloff et al., 2012). Furthermore, one of the important risk factors for the degenerative process of OA is the inflammatory response. Elevated levels of inflammatory mediators have been detected in almost every OA joint tissue (synovium, subchondral bone, and cartilage, etc.; Orlowsky and Kraus, 2015).

PD is a progressive neurodegenerative disease caused by the loss of dopaminergic neurons (Bhat et al., 2018) and is characterized by motor (e.g., resting tremor, muscle stiffness, and bradykinesia) and nonmotor symptoms (e.g., apathy, orthostatic hypotension, and olfactory dysfunction; Modugno et al., 2013; DeMaagd and Philip, 2015). The global prevalence of PD is 0.1%–0.2% (Tysnes and Storstein, 2017), and the prevalence has gradually increased over the past few decades (Aktas et al., 2007; Aid and Bosetti, 2011). PD is often characterized by neuroinflammation, with glia-mediated responses and increased expression of proinflammatory substances (Cebrian et al., 2015).

Recent studies have shown that arthritis is the most common comorbidity of PD (Jones et al., 2012). OA significantly affects the joints of the hip, knee, and spine, resulting in joint inflammation, wear, and stiffness (Loeser et al., 2012; Jacob et al., 2021). Neurologically healthy individuals with lower extremity OA frequently exhibit slower pace, shortened stride length, reduced single-limb support, and reduced mobility compared with healthy controls (Brandes et al., 2008; Zasadzka et al., 2015). Similar to patients with PD, patients with OA also show increases in falls, disease severity, gait dysfunction, and mobility impairment throughout aging (Guideline for the prevention of falls in older persons, 2001; Astephen et al., 2008; Centers for Disease Control and Prevention, 2013). Furthermore, a growing body of literature reports that peripheral inflammation may induce neuroinflammation in the brain, leading to neurodegeneration (Perry, 2004; Träger and Tabrizi, 2013; Wang J. et al., 2018). Therefore, we hypothesized that having OA might increase the risk of developing PD.

In this study, on the basis of previous studies, we used bioinformatics methods to gradually identify common genes related to OA and PD, and preliminarily confirmed the relationship between OA and PD. The correlation between the occurrence and development of diseases, and the correlation between the two in terms of function and immune infiltration were found, thus providing new targets and references for the clinical research of OA and PD in the later stage.

## Materials and Methods

### Data collection

GSE55235 (Woetzel et al., 2014) and GSE7621 (Lesnick et al., 2007) gene expression profiles were obtained from the GEO database^1^ (Edgar et al., 2002), which is a comprehensive microarray and high-throughput sequencing dataset encompassing all research submissions. The two datasets were based on the GPL96 platform (Affymetrix Human Genome U133A Array) and the GPL570 platform (Affymetrix Human Genome U133 Plus 2.0 Array). The GSE55235 dataset contains synovial tissue samples from 10 patients with OA and 10 normal volunteers. The GSE7621 dataset contains 16 samples from Parkinsonian patients with postmortem human substantia nigra and nine samples from normal volunteers.

### Data transformation and visualization

The “limma” R package was used to screen significative genes between OA and PD samples (Ritchie et al., 2015). The DEGs with an adjusted *p* < 0.05 and |log2FC| > 1 were considered statistically significant. Then, the “ggplot2” (Lott et al., 2009) and “pheatmap” R package were used to plot the volcano diagram and heatmap.

### WGCNA-based module and gene screening

To identify common genes linked with OA and PD, the “WGCNA” R package (Langfelder and Horvath, 2008) was used to generate two weighted gene co-expression networks from the two expression matrices. First, merging all the samples ensured a trustworthy network. Second, we computed Pearson correlation coefficients between each pair of genes to measure expression similarity and create a correlation matrix. We also utilized the soft threshold method to create a weighted neighborhood matrix. We utilized a soft connectivity technique to identify the best soft threshold to guarantee gene correlations were scale-free. The neighborhood matrix becomes a topological overlap matrix (TOM). Using dynamic tree cutting and a minimum of 50 genes per module, co-expression modules were produced. Gene significance (GS) and module membership (MM) were computed to link modules to clinical characteristics. Finally, we mapped eigengenes. Venn diagram was carried out by R (version 4.2.0) to overlap the genes between OA and PD.

### Analysis of the enrichment of function in common genes

To get a deeper understanding of the principal biological functions of common genes for OA and PD, we analyzed the GO and KEGG pathways *via* the “ClusterProfiler” R package (Wu et al., 2021), *P* < 0.05 was deemed statistically significant.

### Identification of hub genes in OA-related PD

Search Tool for the Retrieval of Interacting Genes (STRING^2^; version 11.5; Franceschini et al., 2013) may search for interactions between proteins of interest, such as direct binding associations, to form a protein-protein interaction (PPI) network with complicated regulatory linkages. Interactions with a combined score over 0.4 were considered statistically significant. Cytoscape^3^ (version 3.9.1; Shannon et al., 2003) was used to visualize this PPI network of DEGs. And the MCODE (Bader and Hogue, 2003) was executed to build PPI network modules with the following parameters: degree cutoff = 2, node score cutoff = 0.2, k-core = 2, and max. depth = 100. The GeneCards database^4^ was then applied to uncover further information on the hub genes by identifying the related genes, proteins, and disease interactions.

### Validation of hub genes and prediction of diagnostic efficiency

To verify the hub genes, we investigated the expression variation of these genes in other OA and PD datasets (GSE12021 and GSE42966; Huber et al., 2008; Quan et al., 2021). Data from two datasets were compared using “limma” R package to find the DEGs. The cutoff value was |log2FC| >0.8, *P* value < 0.01. Cluster analysis with the R package “pheatmap.” DEGs from the OA and PD datasets were merged using the web tool draw Venn diagram^5^. Moreover, ROC analysis of GSE55235, GSE12021, GSE7621, and GSE42966 was performed to evaluate whether hub genes could differentiate OA and PD samples from their control samples by using the “pROC” R package (Robin et al., 2011).

### Analysis of immune infiltration

Analysis of integrated gene expression data (GSE55235 and GSE7621) using the CIBERSORT algorithm reveals the proportion of 22 immune cell types (Xue et al., 2021). Correlation heatmap was generated after detection of association between immune cells in OA and PD samples with the “corrplot” R package (Serang et al., 2017). Then, the “vioplot” R package was used to illustrate the expression differences of 22 immune cell types in two datasets.

### WDR43 and infiltrating immune cells correlation analysis

The Spearman correlation analysis on WDR43 and Infiltrating immune cells was performed with the use of the “ggpubr,” “ggExtra” and “reshape2” (Zhang, 2016) R packages. The above results were then visualized using the barplot function in R (version 4.2.0).

## Results

### Visualization of data variance analysis results

**Figure 1:**
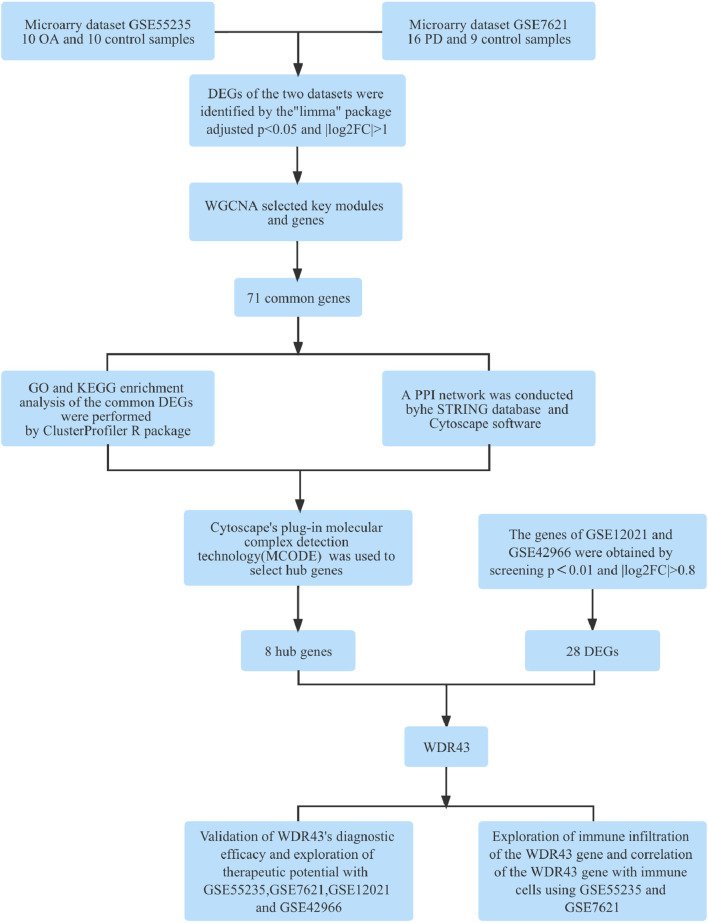
Research design flow chart.

**Figure 2:**
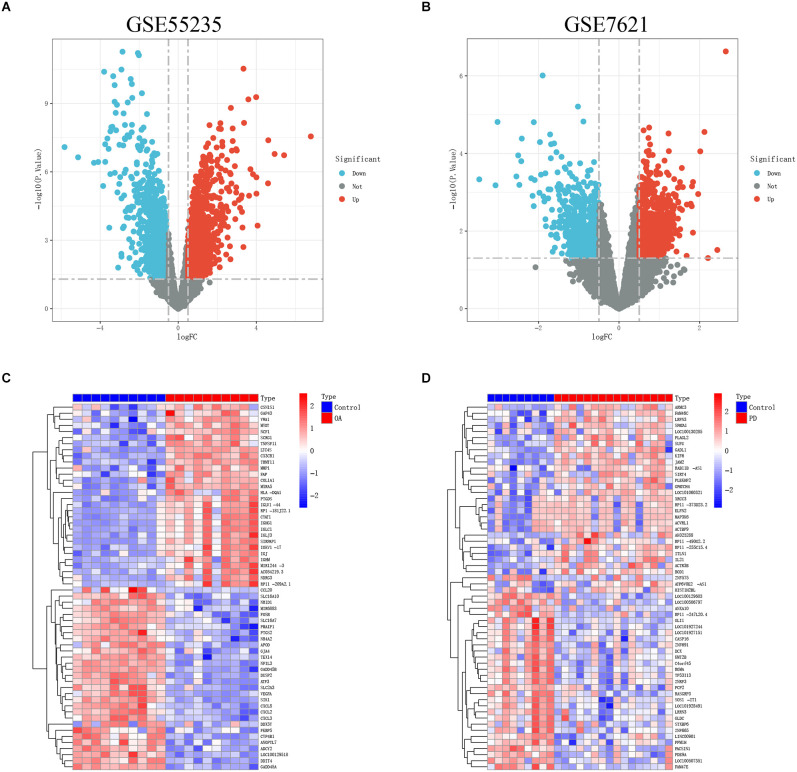
**(A,B)** The volcanoes map of GSE55235 and GSE7621. **(C,D)** The heatmaps of GSE55235 and GSE7621.

Figure 1 depicts the flowchart for this study. After standardizing the microarray results, the “pheatmap” and “ggplot2” R packages were chosen to generate heatmaps and volcano maps of top 30 significantly changed genes (Figures 2A–D).

### Identification of co-expression gene modules

**Figure 3:**
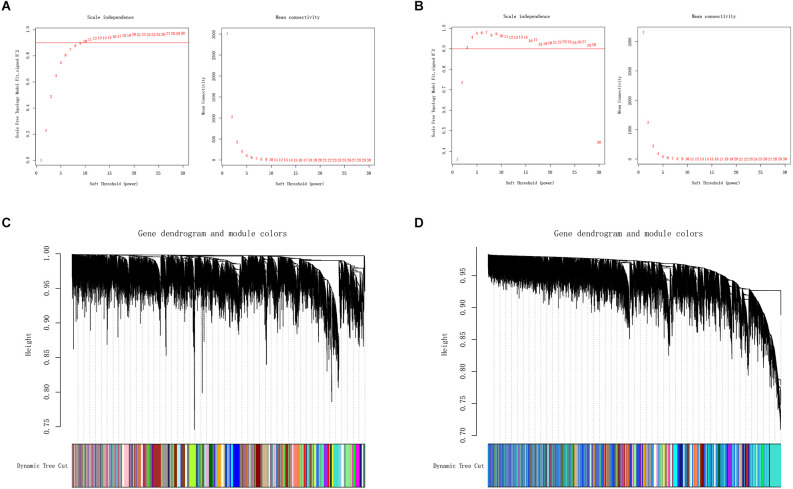
**(A,B)** Definition of soft threshold power through Weighted Gene Co-Expression Network Analysis (WGCNA). Scale-free indices and mean connectivity were analyzed for various soft threshold powers (β). **(C,D)** A gene dendrogram was created by averaging the results of a chain hierarchy clustering algorithm. The colorful rows underneath the tree diagram indicated the assignment of modules based on dynamic tree cutting.

We applied WGCNA to discover gene modules co-expressed in OA and PD datasets. Following the exclusion of sample outliers from both datasets, the remaining data were grouped into control and disease groups. Then, nine and three were selected as the soft threshold power for GSE55235 and GSE7621 based on scale independence greater than 0.90 to assure physiologically significant scale-free networks (Figures 3A,B). Using the dynamic branching cut method on GSE55235 and GSE7621, the genes were put into 40 and 44 modules, respectively (Figures 3C,D).

### Acquisition of common genes

**Figure 4:**
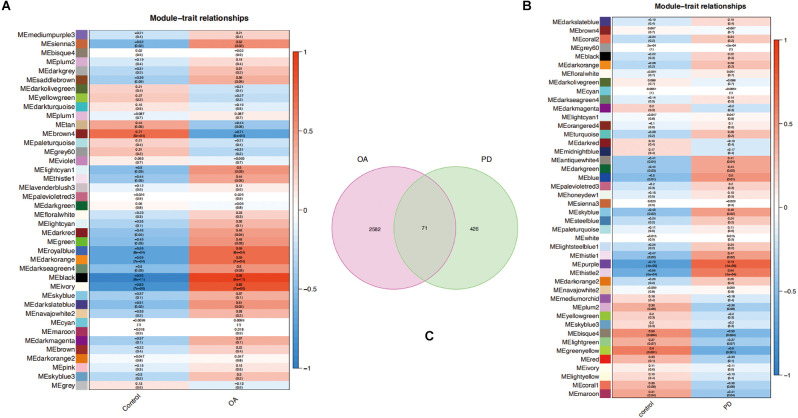
**(A,B)** Each module eigengene (ME) was represented by a row in the heatmaps, and each clinical trait was shown in the columns. **(C)** Venn diagram of 71 common genes in the osteoarthritis (OA) and Parkinson’s disease (PD) datasets.

Key modules related to OA and PD were found by calculating GS and MM to connect modules with clinical characteristics. The association of clinical characteristics in the control and disease groups was represented in the figures. The module eigengene (ME)black and MEivory modules were strongly associated with OA (Figure 4A), whereas the MEpurple and MEthistle2 modules were significantly associated with PD (Figure 4B). Then, we overlapped the genes of those modules to get 71 common genes (Figure 4C).

### Investigation of the biological functions of common genes

**Figure 5:**
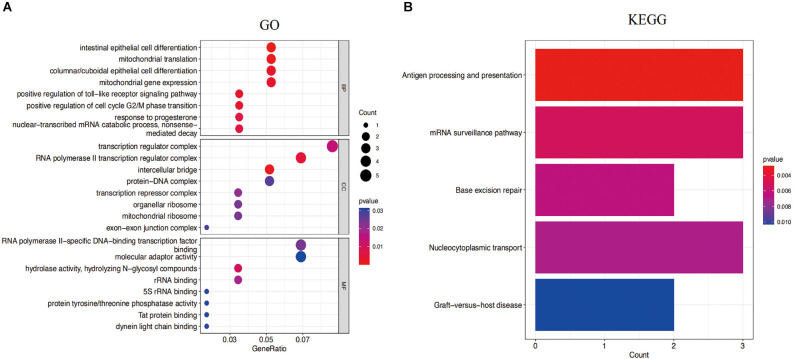
**(A)** Gene Ontology (GO) enrichment results for the top eight common genes. **(B)** Kyoto Encyclopedia of Genes and Genomes (KEGG) pathway result of the top eight common genes. *P*-value < 0.05 was considered significant.

The functional expression distribution of common genes was studied by looking at the GO and KEGG pathways. At first, GO analysis showed these common genes were mainly involved in intestinal epithelial cell differentiation, mitochondrial translation, positive regulation of cell cycle G2/M phase transition, and nuclear-transcribed mRNA catabolic process of the biological process. Moreover, these common genes were mainly associated with the RNA polymerase II transcription regulator complex, transcription regulator complex and intercellular bridge of the cellular component (Figure 5A). Furthermore, the findings of the KEGG analysis demonstrated that these common genes were enriched in antigen processing and presentation, mRNA surveillance pathway, and nucleocytoplasmic transport (Figure 5B).

### Protein-protein interaction (PPI) network analysis of common genes in OA-associated PD

**Figure 6:**
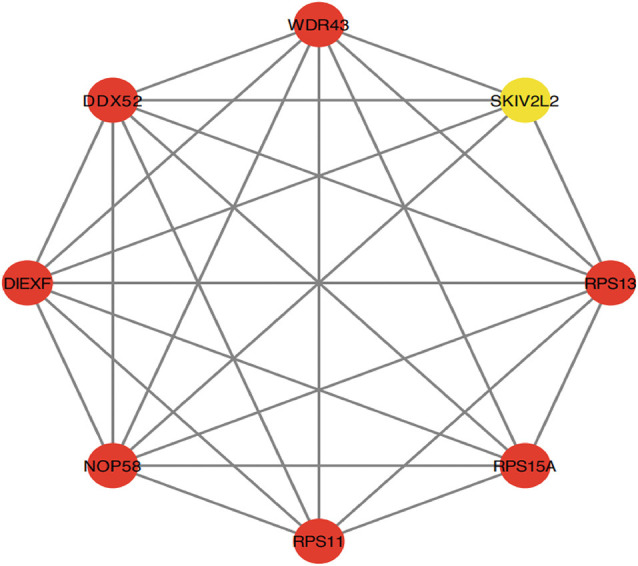
Protein-protein interaction (PPI) network constructed using the STRING database. The eight genes with the most connectivity under Molecular Complex Detection Algorithm (MCODE) were visualized using Cytoscape software.

Cytoscape was used to generate the PPI network of common genes with a composite score larger than 0.4. After we explored the upstream and downstream genes. In the PPI network, there were 124 nodes and 163 edges, where nodes represent genes and edges represent their interactions, and the MCODE algorithm identified eight hub genes, including PRS11, UTP25, RPS15A, RPS13, DDX52, NOP58, SKIV2L2, and WDR43 (Figure 6). Based on the GeneCards database, Table 1 shows their full names and related disorders.

**Table 1:** Details of the hub genes.

**No.**	**Gene symbol**	**Full name**	**Gene-related diseases**
1	RPS11	Ribosomal Protein S11	Cyclosporiasis, Diamond-Blackfan Anemia
2	UTP25	UTP25 Small Subunit Processome Component	Tetraamelia Syndrome
3	RPS15A	Ribosomal Protein S15a	Diamond-Blackfan Anemia 20, Diamond-Blackfan Anemia, Macrocytic Anemia
4	PRS13	Ribosomal Protein S13	Gaucher Disease, Type Ii, Sphingolipidosis, Gaucher’s Disease, Neuroblastoma
5	DDX52	DExD-Box Helicase 52	Chromosome 17q12 Deletion Syndrome
6	NOP58	NOP58 Ribonucleoprotein	Dyskeratosis Congenita
7	SKIV2L2	Ski2 like RNA helicase 2	Trichohepatoenteric Syndrome, Diarrhea, Diarrhea 5, With Tufting Enteropathy, Congenital, Optic Disc Anomalies with Retinal and/or Macular Dystrophy
8	WDR43	WD Repeat Domain 43	3 mc Syndrome, Treacher Collins Syndrome 1

### Validation of the core hub gene

**Figure 7:**
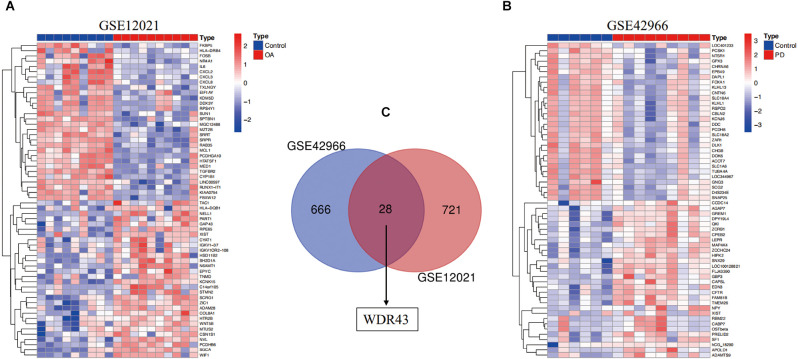
**(A)** A heat map illustrating the hierarchical clustering of differentially expressed genes (DEGs) within the GSE12021 datasets. **(B)** A heat map illustrating the hierarchical clustering of DEGs within the GSE42966 datasets. **(C)** The GSE12021 and GSE42966 datasets were analyzed for the overlap of DEGs. WDR43 were also DEGs in genes shared by the GSE42966 (blue) and GSE12021 (red) datasets.

**Figure 8:**
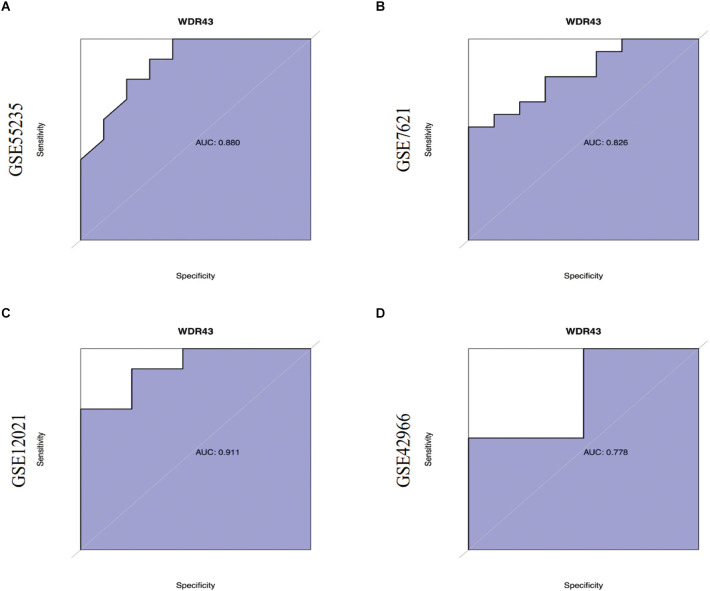
**(A–D)** Receiver Operating Characteristic (ROC) curves of WDR43 in GSE55235, GSE7621, GSE12021, and GSE42966 datasets.

First, we validated hub genes using the GEO datasets GSE12021 for OA and GSE42966 for PD. The clustering heatmaps of DEGs from the GS12021 and GSE42966 datasets were shown in Figures 7A,B. Second, after using Venn diagrams to compare the DEGs from the OA and PD datasets, we found that WDR43, a common gene, was still different (Figure 7C). Moreover, the core hub gene of WDR43 may play a significant role in OA and PD. Finally, this study examined diagnostic performance of WDR43 in four datasets by applying the “pROC” R package, the result as follows: AUC = 0.880 in GSE55235, AUC = 0.826, AUC = 0.911, and AUC = 0.778 (Figures 8A–D).

### Immune infiltration in OA and PD

**Figure 9:**
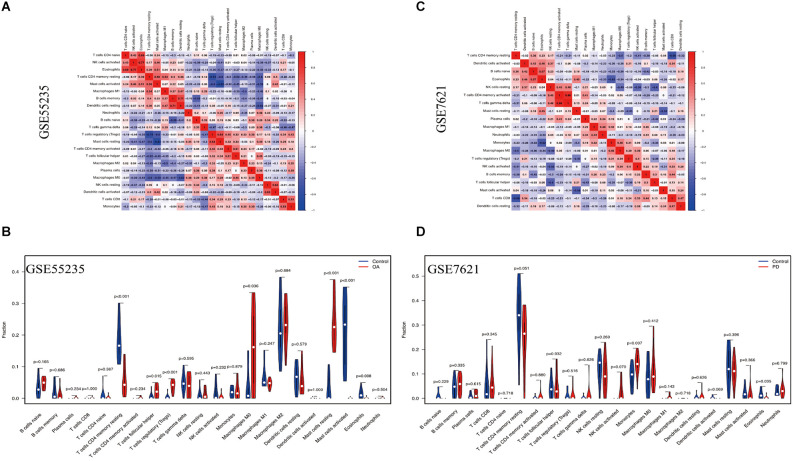
Immune cell infiltration analysis. **(A,B)** The correlation heat map depicted the link between 22 invading immune cells. Red squares indicate positive correlations, while blue squares indicated negative correlations. The size of the squares indicated the strength of the association. **(C,D)** The violin plot showed the distinction of 22 infiltrating immune cells among the OA and PD samples.

The CIBERSORT technique was used to construct the infiltration abundance matrix of 22 immune cell types from the dataset. On the one hand, the heatmap of 22 immune cell types in GSE55235 indicated: Eosinophils and NK cells activated had a crucial positive correlation, T cells CD4 naive were emphatically correlated with Eosinophils, Dendritic cells resting and B cells memory also had a vital positive correlation, and T cells regulatory and Mast cells resting also positively correlated. While T cells CD4 memory resting and T cells regulatory had a passive correction. Mast cells activated and Mast cells resting also negatively correlate (Figure 9A). Moreover, the heatmap of 22 immune cell types included in the dataset GSE7621 revealed that the relationship between B cells naive and Eosinophils was favorable, T cells gamma delta and T cells memory activated had an actively corrected. While T cells CD4 memory resting and T cells CD8 were negatively relevant, eosinophils and monocytes had a passively correlated, and T cells follicular helper and NK cells resting were contrasted (Figure 9B). On the other hand, T cells follicular helper, T cells regulatory, Macrophages M0 and Mast cells resting were highly expressed and significant in the OA group, T cells CD4 memory resting, Mast cells activated and eosinophils were low expression and meaningful in control group (Figure 9C). At the same time, Monocytes demonstrated statistically significant and strong expression in PD groups. Eosinophil immune cells’ expression was low in control groups (Figure 9D).

### Analysis of WDR43 and immune infiltrating cells for correlation

**Figure 10:**
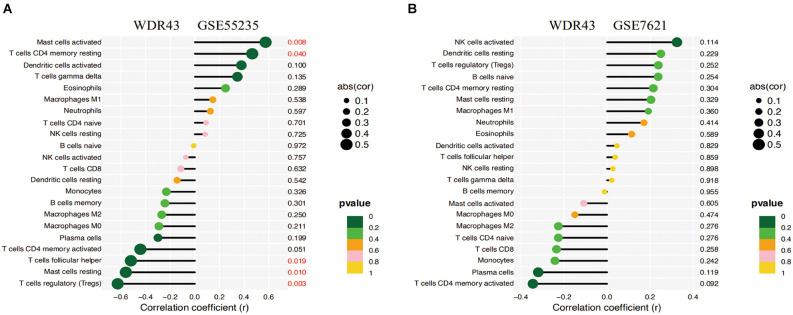
**(A,B)** Correlation analysis of WDR43 and immune infiltrating cells, the size of the circle determined the intensity of the correlation’s absolute value. *P* values less than 0.05 was highlighted in red, with smaller and smaller *P* values progressing from yellow to dark green.

The correlation study indicated that WDR43 was positively connected with Mast cells activated (*r* = 0.574, *p* = 0.008), T cells CD4 memory resting (*r* = 0.466, *p* = 0.040). Moreover, WDR43 was negatively associated with T cells follicular helper (*r* = −0.520, *p* = 0.019), Mast cells resting (*r* = −0.560, *p* = 0.010), and T cells regulatory (*r* = −0.627, *p* = 0.003) in the dataset of GSE55235 (Figures 10A,B).

## Discussion

The current study has initially confirmed that the risk of PD in patients with OA is 41% higher than that in patients without OA. The relationship between OA and PD may involve common risk factors, such as inflammation (Scanzello, 2017; Pajares et al., 2020) and vitamin D deficiency (Zhang et al., 2014; Garfinkel et al., 2017; Sleeman et al., 2017), as well as several mediating factors, such as physical inactivity (Shih et al., 2006; Fang et al., 2018), hypertension (Veronese et al., 2018; Chen et al., 2019), and depression (Veronese et al., 2017; Wang S. et al., 2018).

Recent studies have shown that inflammatory mediators play a key role in the development of OA (Goldring and Otero, 2011; Berenbaum, 2013). Levels of interleukin-6 (IL-6) and tumor necrosis factor-α (TNF-α), both key pro-inflammatory cytokines that induce cartilage catabolism, are elevated in OA patients (Chow and Chin, 2020; Osteoarthritis linked to higher parkinson’s disease risk, 2021). In addition, IL-6 and TNF-α can also activate microglia in the brain to produce pro-inflammatory cytokines in the brain, which may lead to further neuroinflammation in the brain, thereby accelerating neurodegeneration (Dufek et al., 2015; Le et al., 2016; Roper et al., 2020). Studies have found that a large number of microglia are present in the substantia nigra, and that midbrain dopaminergic neurons are particularly susceptible to inflammatory stimulation (Obeso et al., 2010; Tansey and Goldberg, 2010; Teder-Braschinsky et al., 2019). Therefore, we hypothesized that the higher PD risk observed in OA patients might be mediated through cytokine-induced neuroinflammation.

Furthermore, lower vitamin D levels are associated with the development of PD (Lv et al., 2014). Recent studies have shown that vitamin D can promote dopamine synthesis by increasing tyrosine hydroxylase activity. Vitamin D also promotes the production of glial cell-derived neurotrophic factor, a key protein for dopaminergic neuron survival (Pertile et al., 2018). In addition, high levels of vitamin D receptors are found in dopaminergic neurons within the substantia nigra (Cui et al., 2013; Feng et al., 2021). Therefore, chronic insufficiency of vitamin D may accelerate the degeneration of dopaminergic neurons, especially in the substantia nigra region, thereby promoting the development of PD. Studies have shown that patients with OA have lower serum vitamin D levels (Veronese et al., 2015; Bassiouni et al., 2017) and a higher prevalence of vitamin D deficiency (24%–81%; Jansen and Haddad, 2013; Goula et al., 2015). Therefore, OA-related vitamin D deficiency may also increase the risk of PD.

In addition to these two common risk factors, the relationship between OA and PD may involve several mediators, such as physical inactivity, high blood pressure, and depression. Because physical activity may temporarily increase pain and disability, patients with OA are less likely to receive physical activity recommendations than the general population (Herbolsheimer et al., 2016). At the same time, studies have shown that there is a dose-response negative correlation between physical activity and PD, which may be caused by the increased production of various growth factors and reduced oxidative stress caused by physical activity (Fang et al., 2018). Interestingly, some data suggest that OA is also a risk factor for hypertension (Veronese et al., 2018), and hypertensive patients may contribute to PD through basal ganglia hypertensive vascular disease (Chen et al., 2019). Finally, patients diagnosed with OA have a significantly higher risk of depression compared with patients without OA (Veronese et al., 2017). At the same time, there is a significant correlation between depression and the incidence of PD because they share common physiopathological features (e.g., brain atrophy and decreased GABA levels; Wang S. et al., 2018).

Through PPI network analysis of OA-related PD common genes and hub gene identification analysis, we found that WDR43 may play an important role in OA and PD. WDR43 is associated with congenital growth disorder 3-M syndrome, which causes mutations in three proteins (CUL7, OBSL1, and CCDC8), a congenital short stature disorder with a head deformity that exhibits only growth-related defects (Hanson et al., 2014). Some studies have used siRNA and shRNA virus to silence WDR43 to confirm the function of WDR43 in nucleolar fusion. After mitosis, multiple small nucleoli are formed around transcriptionally active NOR. As the cell cycle progresses, these small nucleoli fuse to form larger nucleoli (Hernandez-Verdun et al., 2010; Moore et al., 2022). Although the mechanism of action of WDR43 in nucleolar fusion is unclear, based on the fact that inhibition of NOL11 results in the formation of a large nucleolus, preventing nucleolar fusion may not be a common phenomenon in all ribosomal biogenesis protein mutations (Freed et al., 2012). One possible explanation is that WDR43 deletion might lead to structural changes in rDNA, which in turn might interfere with nucleolar fusion. In addition, studies have shown that WDR43/UTP5 is required for the proper formation of the nucleolus and for the organization and function of the subnucleolus (Sullivan et al., 2001).

We found differential expression of a variety of immune cells through immune infiltration in OA and PD, suggesting that immune regulation plays a crucial role in the occurrence and development of OA and PD. The cells distributed in the OA synovium are mainly T lymphocytes and macrophages, which, together with activated synovial cells, are responsible for the production of cytokines and increased angiogenesis, inducing the secretion of metalloproteinases and proteolytic enzymes, and sustaining cartilage degradation (Kapoor et al., 2011; Wojdasiewicz et al., 2014; Prieto-Potin et al., 2015; Klein-Wieringa et al., 2016). Synovial macrophage activation is a prerequisite for the overexpression of matrix metalloproteinases and an important step prior to the upregulation of cytokine production and subsequent cartilage degradation and destruction (Blom et al., 2007; Haseeb and Haqqi, 2013). *In vitro* experiments have shown that depletion of synovial macrophages can significantly reduce the synthesis and release of MMP-1 and MMP-3, as well as the synthesis and release of IL-1β as well as TNF-α, IL-6, and IL-8 (Bondeson et al., 2006). Synthesis of cartilage-specific collagens (e.g., types II and IX) by chondrocytes is inhibited by IL-1β, while proteolytic enzymes (e.g., MMP-13 and ADAMTS-4) are upregulated (Mengshol et al., 2000; Moore et al., 2022). In addition, lower numbers of T cells and B cells were also detected in synovial mononuclear cell infiltration in patients with OA.

Neurovascular units in PD patients are altered to not only activate innate immune responses but also recruit and activate adaptive responses (Bartels et al., 2008; Brochard et al., 2009). Oxidative modification of specific proteins associated with PD (α-Syn nitration) generates novel epitopes capable of initiating peripherally driven CD4^+^ and CD8^+^ T cell responses (Benner et al., 2008). Furthermore, activated microglia induce the expression of MHC class I molecules in human catecholaminergic neurons, making them susceptible to cell death in the presence of cytotoxic T lymphocytes (Cebrian et al., 2014). Notably, elevated levels of cytokines, including IL-1β, IL-2, IL-6, IFN-γ, and TNF-α, as well as CD4^+^ lymphocyte counts, have been detected in the serum and cerebrospinal fluid of PD patients (Brodacki et al., 2008; Reale et al., 2009). The ratio of CD4^+^ to CD8^+^ lymphocytes and the number of Treg lymphocytes were reduced in patients compared with controls (Baba et al., 2005). In mouse models of PD, in addition to microglia and astrocyte activation, there is a marked infiltration of B cells, CD4^+^ T cells, CD8^+^ T cells, and natural killer cells (Kustrimovic et al., 2019).

In this study, we analyzed immune cell infiltration and core genes that may play key roles in OA and PD tissues. However, our study also has certain limitations. This is a bioinformatics study that has not been experimentally validated. Although the results of some previous studies are consistent with our analysis, the reliability of the results of this study needs further experimental verification. In order to deeply understand the relationship between OA and PD, further animal experiments and clinical studies are very necessary. Through bioinformatics research, we have discovered target gene from the dataset that is simultaneously related to OA and PD. In the following investigations, we will examine the impact of the target gene on the survival and function of brain nigrostriatal dopaminergic neurons and articular chondrocytes, as well as establish the correlation between target gene and these two diseases. Furthermore, by establishing mouse models of PD and OA (control and target gene deficient groups), the key regulatory roles of target genes in the development of the two diseases were clarified. Finally, clinical trials are organized to finally clarify the diagnostic and therapeutic value of the target gene.

## Conclusions

Chronic inflammation can directly or indirectly contribute to the progression of OA and PD. However, therapeutic intervention for OA and PD remains an urgent challenge. Due to the central role of inflammation in OA and PD, immunomodulatory therapy is a major target of current research. But this may not alter the underlying cause of the disease, only by reducing the production of inflammatory mediators, resulting in clinical benefit. In this study, we used a variety of bioinformatics methods to unearth the common genes between the pathogenic mechanisms of OA and PD, thus providing a new research direction for future clinical interventions.

## Data Availability Statement

The datasets presented in this study can be found in online repositories. The names of the repository and accession numbers can be found in the article.

## Author Contributions

JLi, WS, and DL developed a major research plan. HH, MH, and JLiu analyzed data, drew charts, and wrote manuscripts. HH and MH helped collect data and references. All authors contributed to the article and approved the submitted version.

## Funding

This work was supported by Suzhou Science and Technology Project (SLJ2021013) and Natural Science Project of Chengdu Medical College (CYZYB21-07).

## Footnotes

^1^ http://www.ncbi.nlm.nih.gov/geo

^2^ http://string-db.org

^3^ http://www.cytoscape.org

^4^ http://www.genecards.org/

^5^ http://bioinformatics.psb.ugent.be/webtools/Venn/

## Abbreviations

OA: Osteoarthritis
PD: Parkinson’s disease
GEO: Gene Expression Omnibus
WGCNA: Weighted Gene Co-Expression Network Analysis
GO: Gene ontology
KEGG: Kyoto Encyclopedia of Genes and Genomes
MCODE: Molecular Complex Detection Algorithm
DEGs: differentially expressed genes
Tregs: T cell regulatory
TOM: topological overlap matrix
PPI: protein-protein interaction
IL-6: interleukin-6.

## References

[B1] Aid S. Bosetti F. (2011). Targeting cyclooxygenases-1 and -2 in neuroinflammation: therapeutic implications. Biochimie 93, 46–51. 10.1016/j.biochi.2010.09.009 20868723 PMC3008299

[B2] Aktas O. Ullrich O. Infante-Duarte C. Nitsch R. Zipp F. (2007). Neuronal damage in brain inflammation. Arch. Neurol. 64, 185–189. 10.1001/archneur.64.2.185 17296833

[B3] Astephen J. L. Deluzio K. J. Caldwell G. E. Dunbar M. J. Hubley-Kozey C. L. (2008). Gait and neuromuscular pattern changes are associated with differences in knee osteoarthritis severity levels. J. Biomech. 41, 868–876. 10.1016/j.jbiomech.2007.10.016 18078943

[B4] Baba Y. Kuroiwa A. Uitti R. J. Wszolek Z. K. Yamada T. (2005). Alterations of T-lymphocyte populations in Parkinson disease. Parkinsonism Relat. Disord. 11, 493–498. 10.1016/j.parkreldis.2005.07.005 16154792

[B5] Bader G. D. Hogue C. W. (2003). An automated method for finding molecular complexes in large protein interaction networks. BMC Bioinformatics 4:2. 10.1186/1471-2105-4-2 12525261 PMC149346

[B6] Bartels A. L. Willemsen A. T. Kortekaas R. de Jong B. M. de Vries R. de Klerk O.. (2008). Decreased blood-brain barrier P-glycoprotein function in the progression of Parkinson’s disease, PSP and MSA. J. Neural Transm. (Vienna) 115, 1001–1009. 10.1007/s00702-008-0030-y 18265929 PMC2468317

[B7] Bassiouni H. Aly H. Zaky K. Abaza N. Bardin T. (2017). Probing the relation between vitamin D deficiency and progression of medial femoro-tibial osteoarthitis of the knee. Curr. Rheumatol. Rev. 13, 65–71. 10.2174/1573397112666160404124532 27041085

[B8] Benner E. J. Banerjee R. Reynolds A. D. Sherman S. Pisarev V. M. Tsiperson V.. (2008). Nitrated alpha-synuclein immunity accelerates degeneration of nigral dopaminergic neurons. PLoS One 3:e1376. 10.1371/journal.pone.0001376 18167537 PMC2147051

[B9] Berenbaum F. (2013). Osteoarthritis as an inflammatory disease (osteoarthritis is not osteoarthrosis!). Osteoarthritis Cartilage 21, 16–21. 10.1016/j.joca.2012.11.012 23194896

[B10] Bhat S. Acharya U. R. Hagiwara Y. Dadmehr N. Adeli H. (2018). Parkinson’s disease: cause factors, measurable indicators and early diagnosis. Comput. Biol. Med. 102, 234–241. 10.1016/j.compbiomed.2018.09.008 30253869

[B11] Blom A. B. Libregts S. Holthuysen A. E. (2007). Crucial role of macrophages in matrix metalloproteinase-mediated cartilage destruction during experimental osteoarthritis: involvement of matrix metalloproteinase 3. Arthritis Rheum. 56, 147–157. 10.1002/art.22337 17195217

[B12] Bondeson J. Wainwright S. D. Lauder S. Amos N. Hughes C. E. (2006). The role of synovial macrophages and macrophage-produced cytokines in driving aggrecanases, matrix metalloproteinases and other destructive and inflammatory responses in osteoarthritis. Arthritis Res. Ther. 8:R187. 10.1186/ar2099 17177994 PMC1794533

[B13] Brandes M. Schomaker R. Möllenhoff G. Rosenbaum D. (2008). Quantity versus quality of gait and quality of life in patients with osteoarthritis. Gait Posture 28, 74–79. 10.1016/j.gaitpost.2007.10.004 18054233

[B14] Brochard V. Combadière B. Prigent A. Laouar Y. Perrin A. Beray-Berthat V. (2009). Infiltration of CD4^+^ lymphocytes into the brain contributes to neurodegeneration in a mouse model of Parkinson disease. J. Clin. Invest. 119, 182–192. 10.1172/JCI36470 19104149 PMC2613467

[B15] Brodacki B. Staszewski J. Toczyłowska B. Kozłowska E. Drela N. Chalimoniuk M.. (2008). Serum interleukin (IL-2, IL-10, IL-6, IL-4), TNFalpha and INFgamma concentrations are elevated in patients with atypical and idiopathic parkinsonism. Neurosci. Lett. 441, 158–162. 10.1016/j.neulet.2008.06.040 18582534

[B16] Cebrian C. Banerjee R. Reynolds A. D. Sherman S. Pisarev V. M. Tsiperson V. (2014). MHC-I expression renders catecholaminergic neurons susceptible to T-cell-mediated degeneration. Nat. Commun. 5:3633. 10.1038/ncomms4633 24736453 PMC4024461

[B17] Cebrian C. Loike J. D. Sulzer D. (2015). Neuroinflammation in Parkinson’s disease animal models: a cell stress response or a step in neurodegeneration? Curr. Top. Behav. Neurosci. 22, 237–270. 10.1007/7854_2014_356 25293443

[B18] Centers for Disease Control and Prevention (2013). Prevalence of doctor-diagnosed arthritis and arthritis-attributable activity limitation–United States, 20102012. MMWR Morb. Mortal. Wkly. Rep. 62, 869–873. 24196662 PMC4585589

[B19] Chen J. Zhang C. Wu Y. Zhang D. (2019). Association between hypertension and the risk of Parkinson’s disease: a meta-analysis of analytical studies. Neuroepidemiology 52, 181–192. 10.1159/000496977 30726850

[B20] Chow Y. Y. Chin K. Y. (2020). The role of inflammation in the pathogenesis of osteoarthritis. Mediators Inflamm. 23:8293921. 10.1155/2020/8293921 32189997 PMC7072120

[B21] Cui X. Pelekanos M. Liu P. Y. Burne T. H. McGrath J. J. Eyles D. W.. (2013). The vitamin D receptor in dopamine neurons; its presence in human substantia nigra and its ontogenesis in rat midbrain. Neuroscience 236, 77–87. 10.1016/j.neuroscience.2013.01.035 23352937

[B22] DeMaagd G. Philip A. (2015). Parkinson’s disease and its management: part 1: disease entity, risk factors, pathophysiology, clinical presentation and diagnosis. P T 40, 504–532. 26236139 PMC4517533

[B23] Dufek M. Rektorova I. Thon V. Lokaj J. Rektor I. (2015). Interleukin-6 may contribute to mortality in Parkinson’s disease patients: a 4-year prospective study. Parkinsons Dis. 2015:898192. 10.1155/2015/898192 26351617 PMC4553204

[B24] Edgar R. Domrachev M. Lash A. E. (2002). Gene expression omnibus: NCBI gene expression and hybridization array data repository. Nucleic Acids Res. 30:20710. 10.1093/nar/30.1.207 11752295 PMC99122

[B25] Egloff C. Hugle T. Valderrabano V. (2012). Biomechanics and pathomechanisms of osteoarthritis. Swiss Med. Wkly. 142:w13583. 10.4414/smw.2012.13583 22815119

[B26] Fang X. Han D. Cheng Q. Zhang P. Zhao C. Min J.. (2018). Association of levels of physical activity with risk of Parkinson disease: a systematic review and meta-analysis. JAMA Netw. Open 1:e182421. 10.1001/jamanetworkopen.2018.2421 30646166 PMC6324511

[B27] Feng S. H. Chuang H. J. Yeh K. C. Pan S. L. (2021). Association of osteoarthritis with increased risk of Parkinson’s disease: a population-based, longitudinal follow-up study. Arthritis Care Res. (Hoboken). 10.1002/acr.24708. [Online ahead of print]. 34105302

[B28] Franceschini A. Szklarczyk D. Frankild S. Kuhn M. Simonovic M. Roth A.. (2013). STRING v9.1: protein-protein interaction networks, with increased coverage and integration. Nucleic Acids Res. 41, D808–D815. 10.1093/nar/gks1094 23203871 PMC3531103

[B29] Freed E.F. Prieto J. L. McCann K. L. McStay B. Baserga S. J. (2012). NOL11, implicated in the pathogenesis of North American Indian childhood cirrhosis, is required for pre-rRNA transcription and processing. PLoS Genet. 8:e1002892. 10.1371/journal.pgen.1002892 22916032 PMC3420923

[B30] Garfinkel R. J. Dilisio M. F. Agrawal D. K. (2017). Vitamin D and its effects on articular cartilage and osteoarthritis. Orthop. J. Sports Med. 5:2325967117711376. 10.1177/2325967117711376 28680892 PMC5480771

[B31] Goldring M. B. Otero M. (2011). Inflammation in osteoarthritis. Curr. Opin. Rheumatol. 23, 471–478. 10.1097/BOR.0b013e328349c2b1 21788902 PMC3937875

[B32] Goula T. Kouskoukis A. Drosos G. Tselepis A. S. Ververidis A. Valkanis C.. (2015). Vitamin D status in patients with knee or hip osteoarthritis in a Mediterranean country. J. Orthop. Traumatol. 16, 35–39. 10.1007/s10195-014-0322-y 25736606 PMC4348522

[B33] Guideline for the prevention of falls in older persons (2001). American geriatrics society, british geriatrics society and american academy of orthopaedic surgeons panel on falls prevention. J. Am. Geriatr. Soc. 49, 664–672. 11380764

[B34] Hanson D. Stevens A. Murray P. G. Black G. C. Clayton P. E. (2014). Identifying biological pathways that underlie primordial short stature using network analysis. J. Mol. Endocrinol. 52, 333–344. 10.1530/JME-14-0029 24711643 PMC4045235

[B35] Haseeb A. Haqqi T. M. (2013). Immunopathogenesis of osteoarthritis. Clin. Immunol. 146, 185–196. 10.1016/j.clim.2012.12.011 23360836 PMC4015466

[B36] Herbolsheimer F. Schaap L. A. Edwards M. H. Maggi S. Otero Á. Timmermans E. J.. (2016). Physical activity patterns among older adults with and without knee osteoarthritis in six european countries. Arthritis Care Res. (Hoboken) 68, 228–236. 10.1002/acr.22669 26212673

[B37] Hernandez-Verdun D. Roussel P. Thiry M. Sirri V. Lafontaine D. L. (2010). The nucleolus: structure/function relationship in RNA metabolism. Wiley Interdiscip. Rev. RNA 1, 415–431. 10.1002/wrna.39 21956940

[B38] Huber R. Hummert C. Gausmann U. Pohlers D. Koczan D. Guthke R.. (2008). Identification of intra-group, inter-individual and gene-specific variances in mRNA expression profiles in the rheumatoid arthritis synovial membrane. Arthritis Res. Ther. 10:R98. 10.1186/ar2485 18721452 PMC2575612

[B39] Jacob L. Loeser R. F. Goldring S. R. Scanzello C. R. Goldring M. B. (2021). Association between osteoarthritis and the incidence of Parkinson’s disease in the United Kingdom. Clin. Parkinsonism Relat. Disord. 5:100120. 10.1016/j.prdoa.2021.100120 34888519 PMC8637132

[B40] Jansen J. A. Haddad F. S. (2013). Haddad, high prevalence of vitamin d deficiency in elderly patients with advanced osteoarthritis scheduled for total knee replacement associated with poorer preoperative functional state. Ann. R Coll. Surg. Engl. 95, 569–572. 10.1308/003588413x13781990150374 24165338 PMC4311532

[B41] Jones J. D. Malaty I. Price C. C. Okun M. S. Bowers D. (2012). Health comorbidities and cognition in 1948 patients with idiopathic Parkinson’s disease. Parkinsonism Relat. Disord. 18, 1073–1078. 10.1016/j.parkreldis.2012.06.004 22776043 PMC6545886

[B42] Kapoor M. Martel-Pelletier J. Lajeunesse D. Pelletier J. P. Fahmi H. (2011). Role of proinflammatory cytokines in the pathophysiology of osteoarthritis. Nat. Rev. Rheumatol. 7, 33–42. 10.1038/nrrheum.2010.196 21119608

[B43] Klein-Wieringa I. R. de Lange-Brokaar B. J. E. Yusuf E. Andersen S. N. Kwekkeboom J. C. Kroon H. M. (2016). Inflammatory cells in patients with endstage knee osteoarthritis: a comparison between the synovium and the infrapatellar fat pad. J. Rheumatol. 43, 771–778. 10.3899/jrheum.151068 26980579

[B44] Kustrimovic N. Marino F. Cosentino M. (2019). Peripheral immunity, immunoaging and neuroinflammation in Parkinson’s disease. Curr. Med. Chem. 26, 3719–3753. 10.2174/0929867325666181009161048 30306855

[B45] Langfelder P. Horvath S. (2008). WGCNA: an R package for weighted correlation network analysis. BMC Bioinformatics 9:559. 10.1186/1471-2105-9-559 19114008 PMC2631488

[B46] Le W. Wu J. Tang Y. (2016). Protective microglia and their regulation in Parkinson’s disease. Front. Mol. Neurosci. 9:89. 10.3389/fnmol.2016.00089 27708561 PMC5030290

[B47] Lesnick T. G. Huber R. Kupfer P. Pohlers D. Pfaff M. Driesch D.. (2007). A genomic pathway approach to a complex disease: axon guidance and Parkinson disease. PLoS Genet. 3:e98. 10.1371/journal.pgen.0030098 17571925 PMC1904362

[B48] Loeser R. F. Goldring S. R. Scanzello C. R. Goldring M. B. (2012). Osteoarthritis: a disease of the joint as an organ. Arthritis Rheum. 64, 1697–1707. 10.1002/art.34453 22392533 PMC3366018

[B49] Lott G. K. 3rd Johnson B. R. Bonow R. H. Land B. R. Hoy R. R. (2009). g-PRIME: a free, windows based data acquisition and event analysis software package for physiology in classrooms and research labs. J. Undergrad. Neurosci. Educ. 8, A50–A54. 23493932 PMC3592704

[B50] Lv Z. Qi H. Wang L. Fan X. Han F. Wang H. (2014). Vitamin D status and Parkinson’s disease: a systematic review and meta-analysis. Neurol. Sci. 35, 1723–1730. 10.1007/s10072-014-1821-6 24847960

[B51] Ma V. Y. Chan L. Carruthers K. J. (2014). Incidence, prevalence, costs and impact on disability of common conditions requiring rehabilitation in the United States: stroke, spinal cord injury, traumatic brain injury, multiple sclerosis, osteoarthritis, rheumatoid arthritis, limb loss and back pain. Arch. Phys. Med. Rehabil. 95, 986–995.e1. 10.1016/j.apmr.2013.10.032 24462839 PMC4180670

[B52] McDonough C. M. Jette A. M. (2010). The contribution of osteoarthritis to functional limitations and disability. Clin. Geriatr. Med. 26, 387–399. 10.1016/j.cger.2010.04.001 20699161 PMC3529154

[B53] Mengshol J. A. Vincenti M. P. Coon C. I. Barchowsky A. Brinckerhoff C. E. (2000). Interleukin-1 induction of collagenase 3 (matrix metalloproteinase 13) gene expression in chondrocytes requires p38, c-Jun N-terminal kinase and nuclear factor kappaB: differential regulation of collagenase 1 and collagenase 3. Arthritis Rheum. 43, 801–811. 10.1002/1529-0131(200004)43:4<801::AID-ANR10>3.0.CO;2-4 10765924

[B54] Modugno N. Lena F. Di Biasio F. Cerrone G. Ruggieri S. Fornai F. (2013). A clinical overview of non-motor symptoms in Parkinson’s disease. Arch. Ital. Biol. 151, 148–468. 24873924

[B55] Moore H. G. Kahan J. B. Sherman J. J. Z. Burroughs P. J. Donohue K. W. Grauer J. N. (2022). Total shoulder arthroplasty for osteoarthritis in patients with Parkinson’s disease: a matched comparison of 90-day adverse events and 5-year implant survival. J. Shoulder Elbow Surg. 31, 1436–1441. 10.1016/j.jse.2022.01.113 35176495

[B56] Neogi T. (2013). The epidemiology and impact of pain in osteoarthritis. Osteoarthritis Cartilage 21, 1145–1153. 10.1016/j.joca.2013.03.018 23973124 PMC3753584

[B57] Obeso J. A. Rodriguez-Oroz M. C. Goetz C. G. Marin C. Kordower J. H. Rodriguez M. (2010). Missing pieces in the Parkinson’s disease puzzle. Nat. Med. 16, 653–661. 10.1038/nm.2165 20495568

[B58] Orlowsky E. W. Kraus V. B. (2015). The role of innate immunity in osteoarthritis: when our first line of defense goes on the offensive. J. Rheumatol. 42, 363–371. 10.3899/jrheum.140382 25593231 PMC4465583

[B59] Osteoarthritis linked to higher parkinson’s disease risk (2021). Saudi Med. J. 42:921. 34344820 PMC9195551

[B60] Pajares M. Rojo A. I. Manda G. Boscá L. Cuadrado A. (2020). Inflammation in Parkinson’s disease: mechanisms and therapeutic implications. Cells 9:1687. 10.3390/cells9071687 32674367 PMC7408280

[B61] Perry V. H. (2004). The influence of systemic inflammation on inflammation in the brain: implications for chronic neurodegenerative disease. Brain Behav. Immun. 18, 407–413. 10.1016/j.bbi.2004.01.004 15265532

[B62] Pertile R. A. N. Cui X. Hammond L. Eyles D. W. (2018). Vitamin D regulation of GDNF/ret signaling in dopaminergic neurons. FASEB J. 32, 819–828. 10.1096/fj.201700713R 29018141

[B63] Prieto-Potin I. Largo R. Roman-Blas J. A. Herrero-Beaumont G. Walsh D. A. (2015). Characterization of multinucleated giant cells in synovium and subchondral bone in knee osteoarthritis and rheumatoid arthritis. BMC Musculoskelet. Disord. 16:226. 10.1186/s12891-015-0664-5 26311062 PMC4550054

[B64] Quan W. Li J. Jin X. Liu L. Zhang Q. Qin Y.. (2021). Identification of potential core genes in Parkinson’s disease using bioinformatics analysis. Parkinsons Dis. 2021:1690341. 10.1155/2021/1690341 34580608 PMC8464436

[B65] Reale M. Iarlori C. Thomas A. Gambi D. Perfetti B. Nicola M. D.. (2009). Peripheral cytokines profile in Parkinson’s disease. Brain Behav. Immun. 23, 55–63. 10.1016/j.bbi.2008.07.003 18678243

[B66] Ritchie M. E. Phipson B. Wu D. Hu Y. Law C. W. Shi W.. (2015). limma powers differential expression analyses for RNA-sequencing and microarray studies. Nucleic Acids Res. 43:e47. 10.1093/nar/gkv007 25605792 PMC4402510

[B67] Robin X. Turck N. Hainard A. Tiberti N. Lisacek F. Sanchez J.-C.. (2011). pROC: an open-source package for R and S+ to analyze and compare ROC curves. BMC Bioinformatics 12:77. 10.1186/1471-2105-12-77 21414208 PMC3068975

[B68] Roper J. A. Schmitt A. C. Gao H. He Y. Wu S. Schmidt P.. (2020). Coexistent osteoarthritis and Parkinson’s disease: data from the Parkinson’s foundation outcomes project. J. Parkinsons Dis. 10, 1601–1610. 10.3233/JPD-202170 32925102

[B69] Roseti L. Desando G. Cavallo C. Petretta M. Grigolo B. (2019). Articular cartilage regeneration in osteoarthritis. Cells 8:1305. 10.3390/cells8111305 31652798 PMC6912428

[B70] Scanzello C. R. (2017). Role of low-grade inflammation in osteoarthritis. Curr. Opin. Rheumatol. 29, 79–85. 10.1097/BOR.0000000000000353 27755180 PMC5565735

[B71] Serang S. Jacobucci R. Brimhall K. C. Grimm K. J. (2017). Exploratory mediation analysis via regularization. Struct. Equation Modeling 24, 733–744. 10.1080/10705511.2017.1311775 29225454 PMC5720177

[B72] Shannon P. Markiel A. Ozier O. Baliga N. S. Wang J. T. Ramage D.. (2003). Cytoscape: a software environment for integrated models of biomolecular interaction networks. Genome Res. 13, 2498–2504. 10.1101/gr.1239303 14597658 PMC403769

[B73] Shih M. Hootman J. M. Kruger J. Helmick C. G. (2006). Physical activity in men and women with arthritis national health interview survey, 2002. Am. J. Prev. Med. 30, 385–393. 10.1016/j.amepre.2005.12.005 16627126

[B74] Sleeman I. Aspray T. Lawson R. Coleman S. Duncan G. Khoo T. K.. (2017). The role of vitamin D in disease progression in early Parkinson’s disease. J. Parkinsons Dis. 7, 669–675. 10.3233/JPD-171122 28984616 PMC5676984

[B75] Sullivan G. J. Bridger J. M. Cuthbert A. P. Newbold R. F. Bickmore W. A. McStay B. (2001). Human acrocentric chromosomes with transcriptionally silent nucleolar organizer regions associate with nucleoli. EMBO J. 20, 2867–2874. 10.1093/emboj/20.11.2867 11387219 PMC125486

[B76] Tansey M. G. Goldberg M. S. (2010). Neuroinflammation in Parkinson’s disease: its role in neuronal death and implications for therapeutic intervention. Neurobiol. Dis. 37, 510–518. 10.1016/j.nbd.2009.11.004 19913097 PMC2823829

[B77] Teder-Braschinsky A. Märtson A. Rosenthal M. Taba P. (2019). Parkinson’s disease and symptomatic osteoarthritis are independent risk factors of falls in the elderly. Clin. Med. Insights Arthritis Musculoskelet. Disord. 12:1179544119884936. 10.1177/1179544119884936 31700249 PMC6823975

[B78] Träger U. Tabrizi S. J. (2013). Peripheral inflammation in neurodegeneration. J. Mol. Med. (Berl) 91, 673–681. 10.1007/s00109-013-1026-0 23546523

[B79] Tysnes O. B. Storstein A. (2017). Epidemiology of Parkinson’s disease. J. Neural Transm. (Vienna) 124, 901–905. 10.1007/s00702-017-1686-y 28150045

[B81] Veronese N. Maggi S. Noale M. Bolzetta F. Zambon S. Corti M. C.. (2015). Serum 25-Hydroxyvitamin D and osteoarthritis in older people: the progetto veneto anziani study. Rejuvenation Res. 18, 543–553. 10.1089/rej.2015.1671 26540555

[B80] Veronese N. Stubbs B. Solmi M. Smith T. O. Noale M. Cooper C.. (2017). Association between lower limb osteoarthritis and incidence of depressive symptoms: data from the osteoarthritis initiative. Age Ageing 46, 470–476. 10.1093/ageing/afw216 27932358

[B82] Veronese N. Stubbs B. Solmi M. Smith T. O. Noale M. Schofield P.. (2018). Knee osteoarthritis and risk of hypertension: a longitudinal cohort study. Rejuvenation Res. 21, 15–21. 10.1089/rej.2017.1917 28648126 PMC5824487

[B84] Wang S. Mao S. Xiang D. Fang C. (2018). Association between depression and the subsequent risk of Parkinson’s disease: a meta-analysis. Prog. Neuropsychopharmacol. Biol. Psychiatry 86, 186–192. 10.1016/j.pnpbp.2018.05.025 29859854

[B83] Wang J. Song Y. Chen Z. Leng S. X. (2018). Connection between systemic inflammation and neuroinflammation underlies neuroprotective mechanism of several phytochemicals in neurodegenerative diseases. Oxid. Med. Cell. Longev. 2018:1972714. 10.1155/2018/1972714 30402203 PMC6196798

[B85] Woetzel D. Huber R. Kupfer P. Pohlers D. Pfaff M. Driesch D.. (2014). Identification of rheumatoid arthritis and osteoarthritis patients by transcriptome-based rule set generation. Arthritis Res. Ther. 16:R84. 10.1186/ar4526 24690414 PMC4060460

[B86] Wojdasiewicz P. Poniatowski L. A. Szukiewicz D. (2014). The role of inflammatory and anti-inflammatory cytokines in the pathogenesis of osteoarthritis. Mediators Inflamm. 2014:561459. 10.1155/2014/561459 24876674 PMC4021678

[B87] Wu T. Hu E. Xu S. Chen M. Guo P. Dai Z.. (2021). clusterProfiler 4.0: a universal enrichment tool for interpreting omics data. Innovation (Camb) 2:100141. 10.1016/j.xinn.2021.100141 34557778 PMC8454663

[B88] Xue G. Hua L. Zhou N. Li J. (2021). Characteristics of immune cell infiltration and associated diagnostic biomarkers in ulcerative colitis: results from bioinformatics analysis. Bioengineered 2, 252–265. 10.1080/21655979.2020.1863016 33323040 PMC8291880

[B89] Zasadzka E. Borowicz A. M. Roszak M. Pawlaczyk M. (2015). Assessment of the risk of falling with the use of timed up and go test in the elderly with lower extremity osteoarthritis. Clin. Interv. Aging 10, 1289–1298. 10.2147/CIA.S86001 26300633 PMC4535541

[B90] Zhang F. F. Driban J. B. Lo G. H. Price L. L. Booth S. Eaton C. B.. (2014). Vitamin D deficiency is associated with progression of knee osteoarthritis. J. Nutr. 144, 2002–2008. 10.3945/jn.114.193227 25411034 PMC4230211

[B91] Zhang Z. (2016). Reshaping and aggregating data: an introduction to reshape package. Ann. Transl. Med. 4:78. 10.3978/j.issn.2305-5839.2016.01.33 27004225 PMC4779770

